# MicroRNA-323-3p inhibits cell invasion and metastasis in pancreatic ductal adenocarcinoma *via* direct suppression of SMAD2 and SMAD3

**DOI:** 10.18632/oncotarget.7482

**Published:** 2016-02-18

**Authors:** Chunyou Wang, Pian Liu, Heshui Wu, Pengfei Cui, Yongfeng Li, Yao Liu, Zhiqiang Liu, Shanmiao Gou

**Affiliations:** ^1^ Pancreatic Disease Institute, Union Hospital, Tongji Medical College, Huazhong University of Science and Technology, Wuhan 430022, People's Republic of China; ^2^ Cancer Center, Union Hospital, Tongji Medical College, Huazhong University of Science and Technology, Wuhan 430022, People's Republic of China; ^3^ Department of Hepatobiliary Surgery, Union Hospital, Tongji Medical College, Huazhong University of Science and Technology, Wuhan 430022, People's Republic of China

**Keywords:** miR-323-3p, pancreatic ductal adenocarcinoma, SMAD2, SMAD3

## Abstract

Pancreatic ductal adenocarcinoma (PDAC), which accounts for 96% of all pancreatic cancer cases, is characterized by rapid progression, invasion and metastasis. Transforming growth factor-beta (TGF-β) signaling is an essential pathway in metastatic progression and microRNAs (miRNA) play central roles in the regulation of various biological and pathologic processes including cancer metastasis. However, the molecular mechanisms involved in regulation of miRNAs and activation of TGF-β signaling in PDAC remain to be established. The results of this study suggested that miR-323-3p expression in PDAC tissues and cell lines was significantly decreased compared to levels in normal pancreatic tissues and primary cultured pancreatic duct epithelial cells. Further investigation revealed that miR-323-3p directly targeted and suppressed SMAD2 and SMAD3, both key components in TGF-β signaling. Lower levels of miR-323-3p predicted poorer prognosis in patients with PDAC. Ectopic overexpression of miR-323-3p significantly inhibited, while silencing of miR-323-3p increased the migration and invasion abilities of PDAC cells *in vitro*. Moreover, using an *in vivo* mouse model, we demonstrated that overexpressing of miR-323-3p significantly reduced, while knockdown of miR-323-3p enhanced lung metastatic colonization of PANC-1 cells. Furthermore, miR-323-3p-induced TGF-b signaling inhibition and cell motility suppression were partially rescued by overexpressing of Smad2 and Smad3 in PDAC cells. Our findings suggest that re-expression of miR-323-3p might offer a novel therapeutic target against metastasis in patients with PDAC.

## INTRODUCTION

Pancreatic ductal adenocarcinoma (PDAC), which accounts for 96% of all pancreatic cancer cases, is the fourth leading cause of cancer related death in developed countries [1]. Despite advances in surgical and medical therapies, PDAC remains one of the most aggressive tumors with an overall cumulative 5-year survival rate <5% [1]. The high mortality rate is primarily due to the high frequency of metastatic disease; over 80% of patients diagnosed with PDAC present too late for curative treatment due to metastasis [2, 3]. The underlying mechanisms concerning cancer metastasis remains to be fully elucidated and is essential to develop anti-metastatic strategies in the future [4].

Transforming growth factor-beta (TGF-β) signaling plays central roles in multiple biological processes, including embryonic development and cancer progression [5, 6]. Hyperactivation of TGF-β signaling has been reported in most types of tumors [7]. Elevated levels of TGF-β ligands in tumor tissues have been correlated with increased levels of metastatic phenotypes and poorer prognoses in cancer patients [7]. Activation of TGF-β signaling is initiated by ligand-induced oligomerization of serine/threonine kinase receptors, namely TGF-β type I receptor (TβRI) and TGF-β type II receptor (TβRII) [8]; the activated ligand-receptor complex phosphorylates receptor-regulated SMADs (R-SMAD), including transcription factors SMAD2 and SMAD3; once phosphorylated, SMAD2 and SMAD3 bind to SMAD4 and translocate to the nucleus where they regulate expression of target genes in a cell type-dependent manner *via* recruitment of transcriptional coactivators or corepressors [9]. The TGF-β-SMAD pathway promotes cancer progression by regulating multiple stages in the metastatic process, including epithelial-to-mesenchymal transition (EMT). EMT enables dissemination of tumor cells from their primary site into circulation [10] and this pathway has been implicated in the induction of several master regulators of EMT, including Snail, Slug, ZEB2 and FOXC2 [11–15]. However, TGF-β is found to supress expression of Twist to induce the reverse of EMT and thereby promote metastatic colonization [16, 17]. Overactive TGF-β-SMAD2 signaling has been reported to maintain epigenetic silencing of E-cadherin *via* regulation of DNA methylation [18].

Increased expression of TGF-β has been associated with vessel invasion, liver metastasis, advanced tumor stages and shorter survival times in patient with PDAC; conversely, targeting TGF-β signaling in human PDAC cells is found to suppress metastasis and prolong survival in mouse model [19–23]. Therefore, determining the regulatory mechanisms that underlie TGF-β signaling in the metastatic process could provide novel targets for therapeutic interventions in PDAC.

MicroRNAs (miRNAs) function as posttranscriptional regulators of gene expression. They are involved in various cellular processes, including cell proliferation, differentiation and apoptosis, and can regulate the expressions of both oncogenes and tumor suppressor genes [24]. However, the specific role of miRNAs in the regulation of TGF-β signaling and metastasis in PDAC remains to be clarified. Alterations in the profiles of various miRNAs have been reported in several tumors, including PDAC [25]. In this study, we found that miR-323-3p was significantly decreased in PDAC cells compared to normal pancreatic cells, and that downregulation of miR-323-3p promoted invasion and metastatic colonization of the lung. Furthermore, miR-323-3p directly suppressed expression of SMAD2 and SMAD3 leading to inactivation of TGF-β signaling. Overall, our results have suggested that re-expression of miR-323-3p may provide a potential strategy for treating patients with metastatic PDAC.

## RESULTS

### Downregulation of miR-323-3p is correlated with PDAC progression

**Figure 1:**
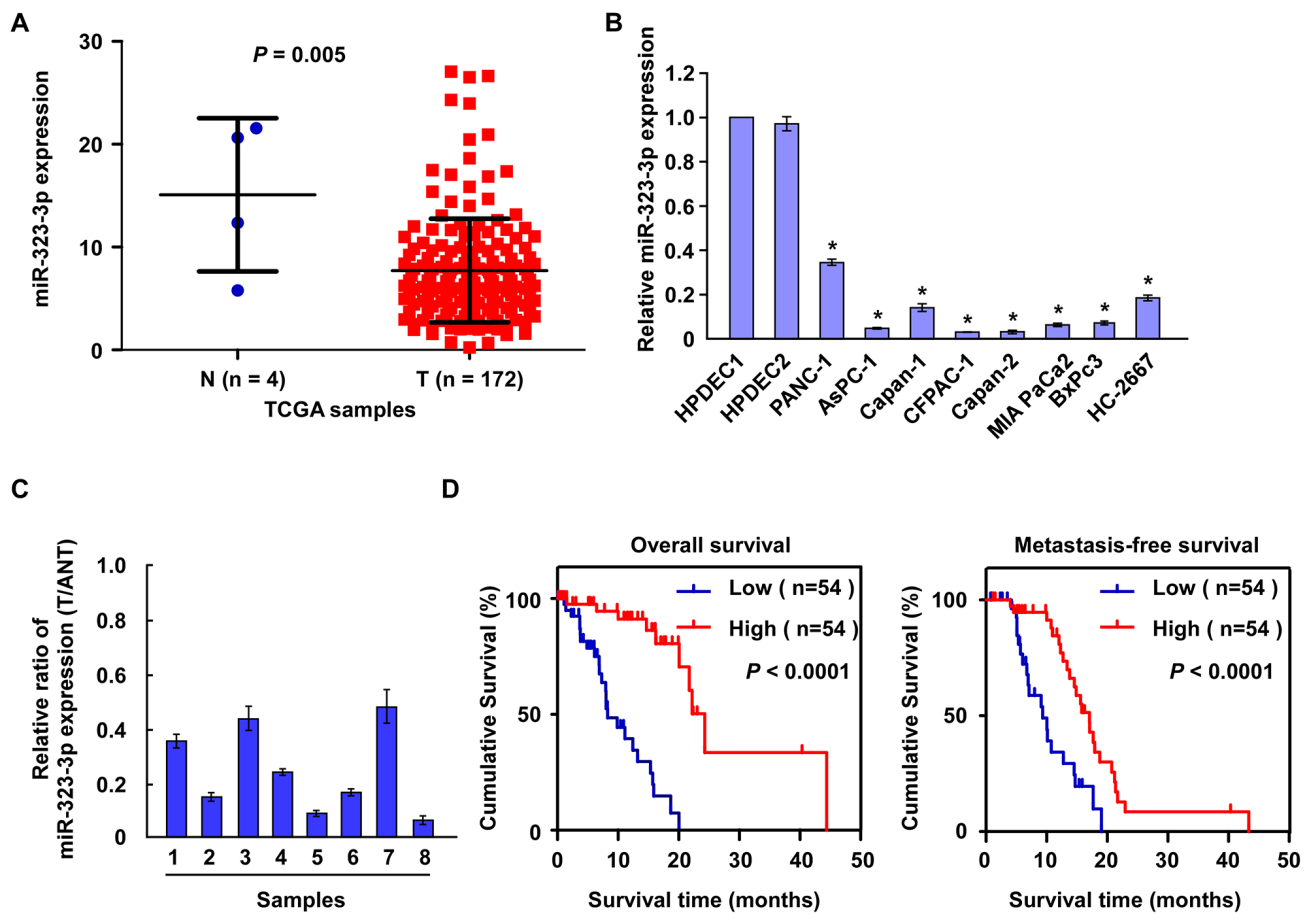
Downregulation of miR-323-3p correlated with PDAC progression **A.** Expression of miR-323-3p was downregulated in PDAC tissues (T; n = 172) compared to normal pancreatic tissues (N; n = 4) based on microarray datasets in The Cancer Genome Atlas (TCGA). **B.** Real-time PCR analysis showed that miR-323-3p expression was downregulated in eight pancreatic cancer cell lines compared to two normal pancreatic cell lines (N). **C.** Real-time PCR analysis showed miR-323-3p expression in eight pancreatic tissues (T) and adjacent normal pancreatic tissues (ANT). Transcript levels were normalized to U6 expression. **D.** Kaplan-Meier survival curves demonstrated that PDAC patients with low miR-323-3p expression (<median; n = 54) had poorer 5-year overall survival (left panel) and metastasis-free survival (right panel) than patients with high miR-323-3p expression (>median; n = 54). Error bars represent the mean ± SD of three independent experiments; * *P* < 0.05.

In order to identify miRNAs involved in the regulation of PDAC progression, miRNA expression profiles were analysed in The Cancer Genome Atlas (TCGA) microarray and GEO datasets. The analyses showed that miR-323-3p was significantly downregulated in pancreatic tumor tissues compared to normal tissues (Figure 1A and Supplementary Figure S1). Consistent with this finding, real-time PCR analyses showed that miR-323-3p expression was significantly downregulated in eight PDAC cell lines relative to two normal pancreatic cell lines (Figure 1B; *P* < 0.05); and was decreased by 2 to 10-fold in eight primary PDAC tissues compared to levels in matched adjacent normal pancreatic tissues (Figure 1C). Comparisons between the expression levels of miR-323-3p in archived PDAC tissue specimens and patients' clinicopathologic characteristics revealed that decreased expression of miR-323-3p was strongly correlated with clinical stage (*P* = 0.046) and TNM classification (T: *P* = 0.037; N: *P* = 0.004; M: *P* = 0.001; Supplementary Table S1 and S2). Furthermore, low expression levels of miR-323-3p predicted shorter overall survival (*P* < 0.001) and metastasis-free survival (*P* < 0.001) in patients with primary PDAC (Figure 1D). Collectively, these results demonstrated that miR-323-3p was downregulated in human PDAC and was associated with enhanced tumor progression and poorer prognosis in patients with PDAC.

### miR-323-3p overexpression inhibits invasion and metastasis of PDAC cells *in vitro* and *in vivo*

**Figure 2:**
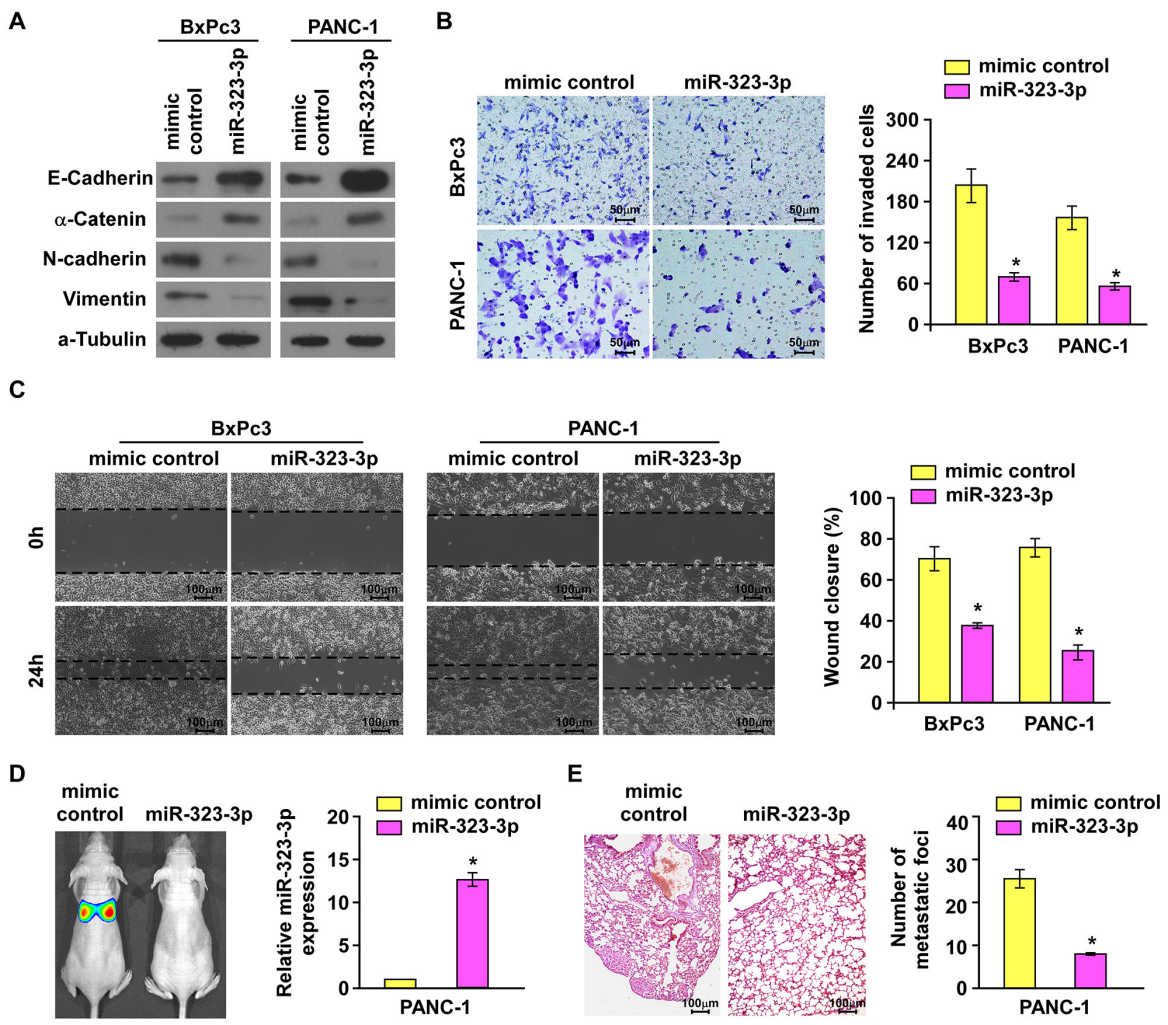
Overexpression of miR-323-3p suppressed invasion and metastasis of PDAC cells *in vitro* and *in vivo* **A.** Western blotting analysis showed the expression levels of epithelial markers (E-cadherin and α-catenin) and mesenchymal markers (N-cadherin and Vimentin) in BxPc3 and PANC-1 PDAC cells transfected with miR-323-3p or vector control. α-tubulin was used as a loading control. **B.** The migratory and invasive properties of BxPc3and PANC-1 cell lines transfected with miR-323-3p or vector control were analyses using a Matrigel-coated Boyden chamber. Magnification, ×400. Error bars represent mean±SD from 3 independent experiments. **C.** Representative micrographs (left panels) and quantification (right panel) of the wound healing assay. The histograms represented the percentage area of recovery 24 h after the initial wounds were created. Magnification, ×100. **D.** Representative images (left panel) and real-time PCR quantification (right panel) showed tumor colonization in the lungs of mice injected with miR-323-3p-transfected PDAC cells or vector control. **E.** Representative micrographs using a dissection microscope showed metastatic lung lesions by H&E staining in the mouse model (left panel). Magnification, ×100. Quantification showing the numbers of lung metastatic foci in the different groups (right panel). Error bars represent the mean ± SD of three independent experiments; * *P* < 0.05.

The biological function of miR-323-3p in the progression of PDAC was investigated by stably transducing miR-323-3p into BxPc3 and PANC-1 PDAC cell lines (Supplementary Figure S2A). Western blotting showed that overexpression of miR-323-3p led to marked upregulation of epithelial markers E-cadherin and a-catenin with concurrent downregulation of mesenchymal markers vimentin and N-cadherin (Figure 2A). Matrigel-coated transwell assays and wound healing assays demonstrated that overexpression of miR-323-3p significantly decreased the invasiveness and migratory capabilities of BxPc3 and PANC-1 cells (Figure 2B–2C; *P* < 0.05) compared to normal control cells. In addition, we also examined the effect of miR-323-3p on cell viability. Anoikis assays showed that miR-323-3p did not affect cell viability of PDAC cells (Supplementary Figure S2B).

Since miR-323-3p did not affect the anoikis resistance of pancreatic cancer cells, we examined the effect of miR-323-3p overexpression on the metastatic colonization capacities of PDAC cells by an intravenously injection mouse model. As shown in Figure 2D and 2E, the number of metastatic lung foci was significantly reduced in mice injected with PANC-1-miR-323-3p-transduced cells compared to those injected with vector control cells. This indicated that metastatic lung colonization could be inhibited by overexpression of miR-323-3p in PDAC cells. Collectively, these results demonstrated that overexpression of miR-323-3p could inhibit PDAC cell motility *in vitro* and abrogated the metastatic progression of PDAC *in vivo*.

### Inhibition of miR-323-3p by antago-miR323-3p promotes invasion and metastasis of PDAC cells *in vitro* and *in vivo*

**Figure 3:**
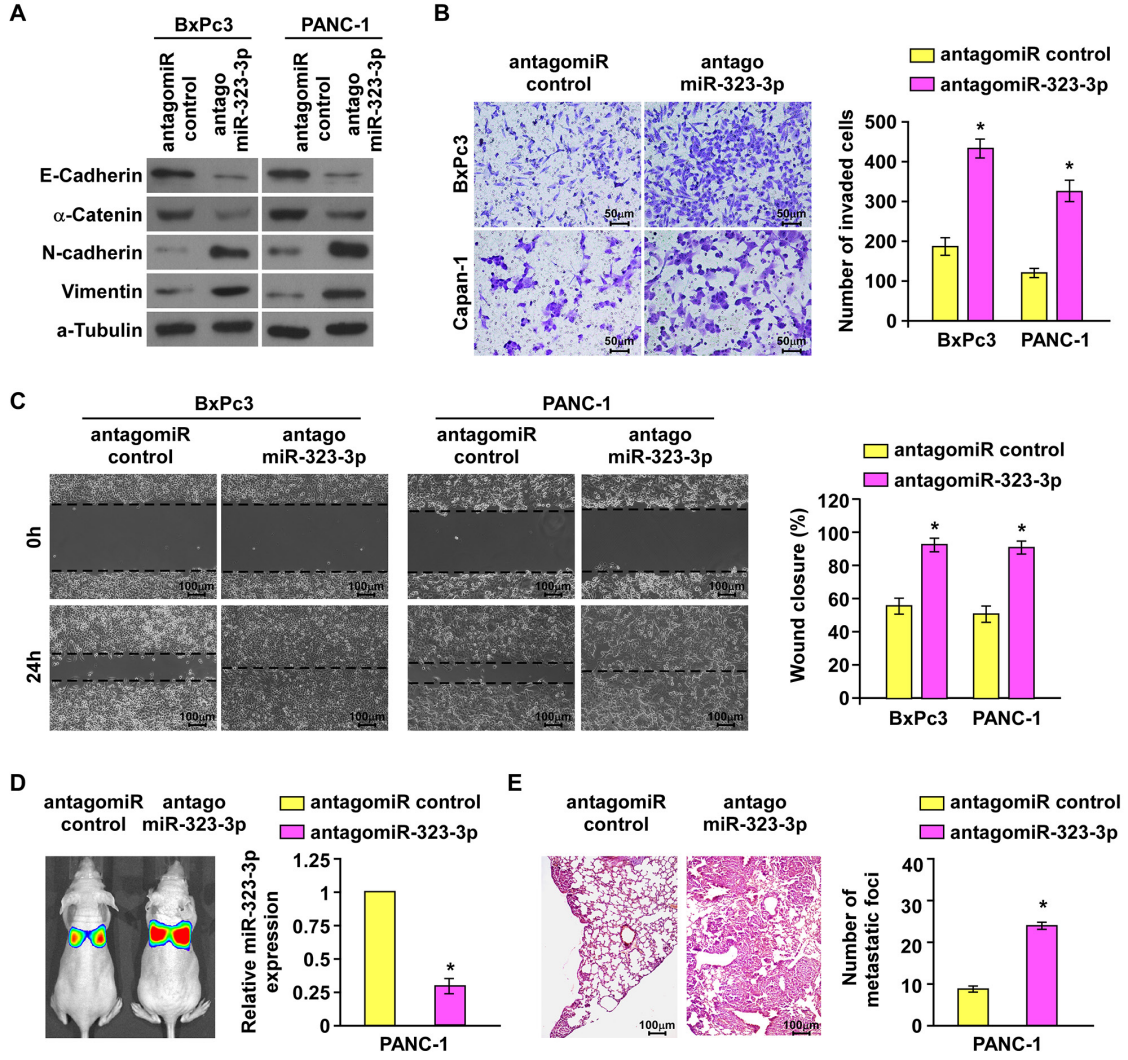
Silencing of miR-323-3p enhanced the invasive and metastatic abilities of PDAC cells *in vitro* and *in vivo* **A.** Western blotting analysis showed the expression levels of epithelial markers (E-cadherin and α-catenin) and mesenchymal markers (N-cadherin and Vimentin) in the indicated cells following silencing of miR-323-3p. α-tubulin was used as a loading control. **B.** and **C.** Matrigel invasion and migration assays (B) and wound healing assays (C) showed enhanced abilities of invasion and migration in BxPc3 and PANC-1 cells following inhibition of miR-323-3p. Magnification, ×400. **D.** Representative images (left panel) and real-time PCR quantification (right panel) showed the levels of tumor colonization in the lungs of mice injected with miR-323-3p-silenced PDAC cells or vector control. **E.** Representative H&E staining images using a dissection microscope showed metastatic lesions in the lungs of mice injected with miR-323-3p-silenced PDAC cells or vector control (left panel). Histograms showed the numbers of metastatic lung foci (right panel). Magnification, ×100. Error bars represent mean ± SD of three independent experiments; * *P* < 0.05.

Having demonstrated that overexpression of miR-323-3p inhibited metastasis in PDAC *in vitro* and *in vivo*, the effect of inhibiting miR-323-3p expression on the invasive and metastatic behaviors of PDAC cells was examined. The expression of miR-323-3p was inhibited by transfection with antago-miR323-3p in BxPc3 and PANC-1 cells (Supplementary Figure S3A). Inhibition of miR-323-3p in BxPc3 and PANC-1cells induced a dramatic morphological change which from cobblestone-like, epithelial morphology into spindle-like, fibroblastic appearance accompanied by a decreased cell-to-cell adhesion (Supplementary Figure S3B). Western blotting confirmed that inhibition of miR-323-3p led to a marked decrease in the levels of epithelial markers E-cadherin and a-catenin with increased levels of mesenchymal markers vimentin and N-cadherin in BxPc3 and PANC-1 PDAC cells compared to their levels in vector control cells (Figure 3A). The invasive and migratory capabilities of BxPc3 and PANC-1 cells were also significantly enhanced by inhibition of miR-323-3p (Figure 3B and 3C; *P* < 0.05). These *in vitro* results were supported by observations *in vivo*: silencing of miR-323-3p in PANC-1 cells injected into a mouse model significantly increased metastatic lung colonization relatively to controls (Figure 3D and 3E; *P* < 0.05; Supplementary Figure S3C). Collectively, these results demonstrated that downregulation of miR-323-3p promoted invasion- and metastasis-related properties of PDAC cells *in vitro* and *in vivo*.

### MiR-323-3p directly suppresses SMAD2 and SMAD3 in PDAC cells

**Figure 4:**
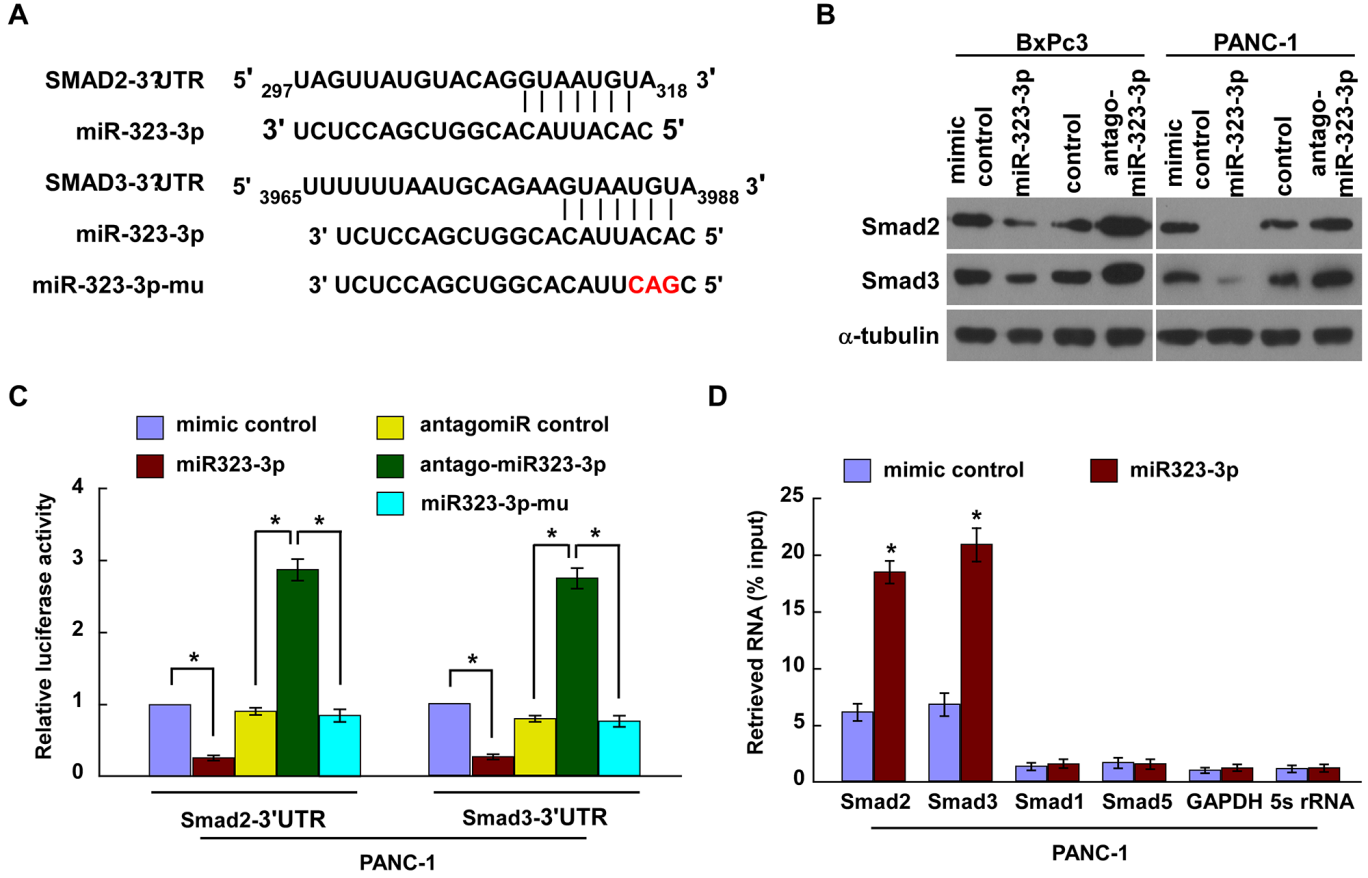
MiR-323-3p directly targeted SMAD2 and SMAD3 **A.** Predicted miR-323-3p target sequences in the 3′UTRs of SMAD2 and SMAD3; the seed sequence of miR-323-3p and a mutant containing three altered nucleotides (miR323-3p-mu). **B.** Western blotting showed the expression levels of SMAD2 and SMAD3 in miR-232-3p-transfected and silenced PDAC cells and their respective controls. α-tubulin was used as a loading control. **C.** Luciferase assay showed the relative activity of targeted SMAD2 and SMAD3 3′UTRs in the indicated cells. **D.** MiRNP immunoprecipitation assay showed the associations between miR-323-3p and SMAD2 and SMAD3 transcripts. SMAD1, SMAD5, GAPDH and 5s rRNA were used as negative controls. Each bar represents the mean ± SD of three independent experiments; *P < 0.05.

TGF-β signaling has been implicated in PDAC metastasis [19–21]. Two key contributors in the TGF-β signaling pathway are SMAD2 and SMAD3 [9]. Exploration of the public bioinformatics tool, TargetScan, predicted that both these proteins were potential targets of miR-323-3p (Figure 4A). In order to explore the regulatory mechanisms by which miR-323-3p promoted PDAC progression, Western blotting and luciferase reporter assays were conducted. Western blotting revealed that expression of SMAD2 and SMAD3 proteins were markedly decreased in miR-323-3p-transduced PDAC cells, whereas inhibition of miR-323-3p had the opposite effect (Figure 4B). Luciferase assays showed that the reporter activities linked with the 3′UTRs of SMAD2 and SMAD3 transcripts were significantly reduced by overexpression of miR-323-3p; whereas, they were increased by inhibition of endogenous miR-323-3p (Figure 4C; *P* < 0.05). Moreover, the suppressive actions of miR-323-3p could be mitigated through a mutation in the seed sequence of miR-323-3p (Figure 4C). Further investigation by immunoprecipitation of microribonucleoprotein (miRNP) revealed that miR-323-3p overexpression enriched the transcripts of SMAD2 and SMAD3, but not that of GAPDH or 5s rRNA controls (Figure 4D). In addition, RIP assay was performed and showed that miR-323-3p did not bind to 3`UTR of Smad1 and Smad5 in the PDAC cell lines (Figure 4D). Consistently, western blotting analysis revealed that overexpression or knockdown of miR-323-3p did not affect the protein expression of Smad1or Smad5 (Supplementary Figure S4), suggesting that Smad1 and Smad5 might not be the authentic targets of miR-323-3p in PDAC cells. Collectively, these results demonstrated that miR-323-3p directly associated with the mRNA 3′UTR regions of SMAD2 and SMAD3 transcripts, thereby identifying SMAD2 and SMAD3 as bona fide targets of miR-323-3p.

### The TGF-β-induced luciferase activity is significantly suppressed in pancreatic cancer cells following overexpression of miR-323-3p

**Figure 5:**
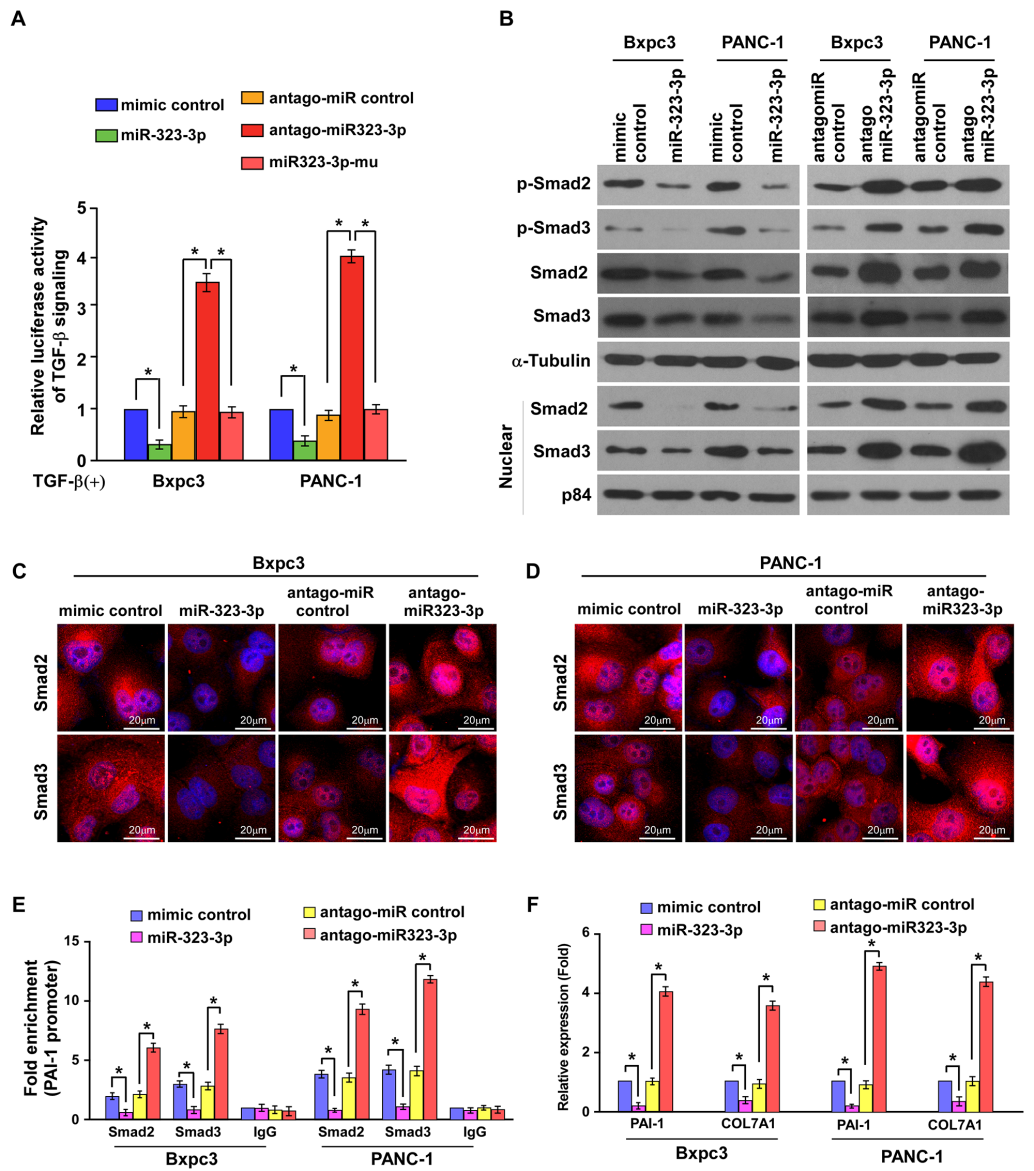
MiR-323-3p inactivates the TGF-β signaling pathway **A.** Luciferase assay showed suppression of TGF-β-induced p3TP-lux transcriptional activity by miR-323-3p in PDAC cells. Each bar represents the mean ± SD of three independent experiments; *P < 0.05. **B.** Western blotting showed the expression levels of total SMAD2, SMAD3, p-SMAD2 and p-SMAD3 and nuclear SMAD2 and SMAD3 in miR-323-3p transfected and silenced PDAC cells. α-tubulin was used as a loading control; nuclear protein p84 served as a nuclear protein marker. **C.** and **D.** Immunofluorescence staining of Smad2 and Smad3 in the Bxpc3 overexpressing or knockdown cells (C) and PANC-1 overexpressing or knockdown cells (D). Scale bars: 20 μm. **E.** ChIP analysis showed the levels of the indicated factors recruited to the *PAI-1* promoter in miR-323-3p-overexpressing and miR-323-3p-knockdown cells. IgG served as a negative control. **F.** PAI-1 and COL7A1 expression in the indicated cell lines was assayed by real-time qRT-PCR. *P < 0.05.

Phosphorylation and subsequent translocation of SMAD2 and SMAD3 to the nucleus are critical steps in TGF-β signal transduction [9]. Therefore, the effect of miR-323-3p in the activation of the TGF-β pathway was examined. The TGF-b-induced luciferase activity was significantly suppressed in PDAC cells following overexpression of miR-323-3p; whereas silencing of miR-323-3p elevated luciferase activity in these cells (Figure 5A; *P* < 0.05). Western blotting revealed that the total expression, phosphorylation and nuclear accumulation of SMAD2 and SMAD3 were reduced by overexpression of miR-323-3p but increased when miR-323-3p expression was silenced (Figure 5B). This was further confirmed by immunofluorescence staining (Figure 5C and 5D). Moreover, the binding affinity of Smad2 and Smad3 to the PAI-1 promoter was significantly decreased in miR-323-3p-transduced cells, while increased in miR-323-3p knockdown cells (Figure 5E). Consistently, the mRNA expression levels of Smad2/3 target genes PAI-1 and COL7A1were decreased in miR-323-3p overexpressing cells but increased in miR-323-3p knockdown cells as determined by Real-time PCR (Figure 5F). In addition, we also showed that miR-323-3p did not significantly alter the activities of AKT, MAPK and nuclear factor of activated T-cells (NFAT) signalling pathways (Supplementary Figure S5A–S5D), which have been involved in the Smad-independent arm of TGF-bβ signalling [26, 27]. In combination, these findings demonstrated that downregulation of miR-323-3p promoted activation of the TGF-β signaling pathway.

### SMAD2 and SMAD3 are functional effectors for the biological functions of miR-323-3p in PDAC cells

**Figure 6:**
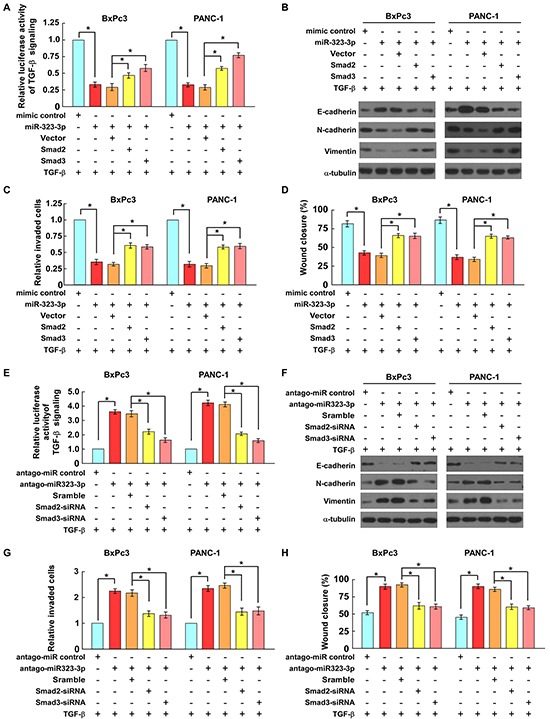
SMAD2 and SMAD3 are functionally important for miR-323-3p-induced cell motility and invasiveness **A.** Luciferase assays showed that expression of SMAD2 or SMAD3 reversed the suppressive effects of miR-323-3p on TGF-β-induced p3TP-lux transcriptional activities in PDAC cells. **B.** The expression levels of EMT markers in miR-323-3p-transduced cells with SMAD2 or SMAD3 overexpression were analyzed by western blotting. **C.** and **D.** Expression of SMAD2 or SMAD3 antagonized the suppressive effects of miR-323-3p on the TGF-β-induced invasiveness (C) and migratory ability (D) of PDAC cells. **E.** Conversely, depletion of SMAD2 or SMAD3 reduced the effects of miR-323-3p-silencing on TGF-β-induced p3TP-lux transcriptional activity in PDAC cells. **F.** The expression levels of EMT markers in miR-323-3p-silencingcells with SMAD2 or SMAD3 knockdown were analyzed by western blotting. **G.** and **H.** Silencing of SMAD2 or SMAD3 inhibited TGF-β-induced cell invasion (G) and migration (H) in miR-323-3p-silencing PDAC cells. Each bar represents the mean ± SD of three independent experiments; *P < 0.05.

The functional roles of SMAD2 and SMAD3 in miR-323-3p-induced TGF-β signaling and the invasive properties of PDAC cells were investigated further in *SMAD4*-wild type PANC-1 and *SMAD4*-null BxPc3 PDAC cell lines [28]. The results showed that the TGF-b-induced transcriptional activities in miR323-3p-transduced cells were significantly decreased as compared to the control cells, which were rescued by ectopic overexpression of the SMAD2 or SMAD3 in these cells (Figure 6A; Supplementary Figure S6A; *P* < 0.05). Furthermore, overexpressing of miR323-3p in PANC-1 and BxPc3 cells significantly inhibited the TGF-b-induced EMT and cell migration/invasion abilities compared to the control cells, while these inhibition effects were partially abrogated by ectopic overexpression of the SMAD2 or SMAD3 (Figures 6B–6D; *P* < 0.05). Conversely, silencing of miR-323-3p in BxPc3 and PANC-1 cells promoted the TGF- b-induced luciferase activities, EMT and cell migration/invasion abilities compared to the control cells, while silencing of SMAD2 or SMAD3 led to a significant reduction of these effects in antagomiR-323-3p transfected cells (Figures 6E-6H and Supplementary Figure S6B; *P* < 0.05). Taken together, these results demonstrated that miR-323-3p-mediated TGF-b signaling inhibition, EMT and cell motility suppression were at least partially caused by repressing of Smad2 and Smad3 in PDAC cells.

### Clinical relevance of miR-323-3p, SMAD2, SMAD3 expression in PDAC

**Figure 7:**
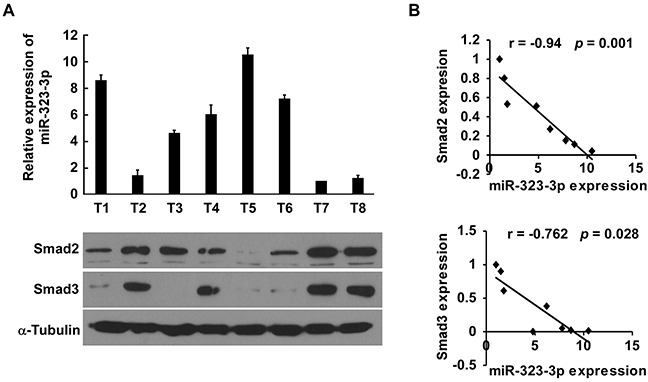
Clinical correlation between miR-323-3p and SMAD2 and SMAD3 in PDAC cells **A.** Expression profiles of miR-323-3p (top) and expression levels of SMAD 2 and SMAD3 protein (bottom) in eight freshly collected human PDAC tissue samples (T). The expression of miR-323 was examined using Real-time PCR analysis and normalized by U6 expression. The sample T7 was used as a standard, and the fold change of miR-323 expression in other samples was calculated by comparing the miR-323/U6 ratio in sample T7. α-tubulin was used as a loading control; each bar represents the mean ± SD of three independent experiments. **B.** Correlation between expression of miR-323-3p and expression of SMAD2 and SMAD3 in the PDAC tissue samples.

Finally, we examined whether miR-323-3p-mediated Smad2 and Smad3 suppression in PDAC is clinically relevant. The expression levels of miR-323-3p, Smad2 and Smad3 were analysed in eight freshly collected PDAC tissue samples (Figure 7A). Correlation studies showed that miR-323-3p levels were inversely correlated with SMAD2 (r = −0.94; *P* = 0.001) and SMAD3 (r = −0.762; *P* = 0.028) protein levels (Figure 7B). This suggested that downregulation of miR-323-3p with increased expression of SMAD2 and SMAD3 contributed to more aggressive phenotypes, leading to worse survival in patients with PDAC. As such, miR-323-3p-mediated suppression of SMAD2 and SMAD3 could be clinically important in PDAC treatments.

## DISCUSSION

The roles of miRNAs in the progression of PDAC are becoming recognized [29, 30] and there is increasing interest in identifying the key miRNAs involved in aggressive phenotypes in PDAC to improve diagnosis, predict prognosis and develop new therapeutic strategies [31]. Several miRNAs have been found to be differentially expressed in tumoral and normal pancreatic tissues [32–34]: decreased expression of miR96 promotes cell invasion and tumor growth in PDAC [35]; low levels of mir-146a induces the metastatic phenotype; whereas high levels of miR-10a promotes invasiveness in PDAC cells [36, 37]. However, prior to this investigation, deregulation of miR323-3p in PDAC was unreported to our knowledge.

Various studies have demonstrated that TGF-β signaling can play contradictory roles in tumor progression: TGF-β inhibits growth in normal and premalignant epithelial cells; whereas, tumor cells become insensitive to the growth-inhibitory effects of TGF-β signaling by accumulating multiple genetic and epigenetic alterations. Instead tumor cells utilize TGF-β to promote EMT and metastasis [38–40]. Mutation or deletion of *SMAD4* gene has been reported in 60% of PDAC cases and has been associated with poor prognosis [41]. Studies have shown that downregulation of SMAD4 could counteract TGF-β-induced cell cycle arrest and promote EMT, resulting in increased motility and invasiveness of cancer cells [26, 42]. These observations suggest that compensatory mechanisms may contribute to the activation of TGF-β signaling and its pro-metastatic effects in PDAC cells.

MiRNAs have the ability to suppress multiple target genes. Therefore enhanced expression of tumor-suppressive miRNAs and inhibition of oncogenic miRNAs may offer more effective treatment strategies than conventional approaches that target single genes alone [25]. Our results showed that restoring miR323-3p expression in PDAC cells could significantly inhibit cell motility *in vitro* and reduce lung metastasis *in vivo*, suggesting that mechanisms capable of promoting or restoring expression of miR-323-3p could be used to suppress malignant progression of PDAC. Further investigation into the underlying mechanisms found that restoring expression of SMAD2 and SMAD3 increased TGF-β-stimulated transcriptional activity of TGF-β signaling and promoted motility and invasion in both *SMAD4*-wild type PANC-1 and *SMAD4*-null BxPc3 PDAC cell lines. Conversely, downregulation of miR323-3p led to nuclear accumulation of SMAD2 and SMAD3 in *SMAD4*-null BxPc3 PDAC cells. This observation was consistent with a previous report that demonstrated that TGF-β could induce nuclear localization of SMAD2 and SMAD3 in SMAD4-null cancer cell lines [43]. Together, these results suggested that decreased expression of miR323-3p contributed to TGF-β-induced aggressive phenotypes in PDAC *via* modulation of SMAD2 and SMAD3 expression.

In summary, to our knowledge, this study has provided the first evidence to identify miR-323-3p as a critical mediator in the activation of TGF-β signaling, suggesting that the mir323-3p/TGF-β signaling cascade may be an important regulatory mechanism in the metastatic progression of PDAC. Our findings may offer potentially novel strategies for therapeutic interventions in the treatment of patients with metastatic PDAC.

## MATERIALS AND METHODS

### Cell culture

Human pancreatic cancer cell lines were purchased from American Type Culture Collection and maintained in a 5% CO2 humidified incubator in DMEM (PANC-1, AsPC-1, CFPAC-1, MIA PaCa-2, BxPc3 and HC-2667) or IMDM (Capan-1 and Capan-2) with 10% (fetal bovine serum) FBS. Primary cultures of normal human pancreatic duct epithelial cells (HPDEC) were established from two fresh specimens of the adjacent non-tumor pancreatic tissues, located more than 5 cm from the cancerous tissues, according to a previous report [44, 45], and maintained in bronchial epithelial basal medium (BEBM; Lonza Walkersville, Walkersville, MD) containing 10% FBS and supplemented with BEGM Single-Quots (Lonza Walkersville).

### Patient information and tissue specimens

A total of 108 freshly-collected surgical specimen of PDAC samples were obtained from the First Affiliated Hospital of Sun Yat-sen University and Sun Yat-sen University Cancer Center from 2005 to 2009. Prior patient consent and approval from the Institutional Research Ethics Committee were obtained for the use of these clinical materials for research purposes. Clinical information on the samples is summarized in Supplementary Table S1. The expression of miR-323-3p was determined by real-time PCR analysis. The median value of miR-323-3p expression in the 108 samples was defined as the cutoff value to classify low and high expression of miR-323. The value ≥ median was defined as high expression, and value < median as low expression. Eight pairs of primary PDAC and adjacent normal tissues were frozen and stored in liquid nitrogen until further use.

### Vectors, retroviral infection and transfection

The human miR-323-3p gene was PCR-amplified from genomic DNA and cloned into a pMSCV-puro retroviral vector. pMSCV-miR-323-3p was co-transfected with the pIK packaging plasmid in HEK293T cells using the standard calcium phosphate transfection method. Thirty-six hours after the cotransfection, supernatants were collected and incubated with PDAC cells to be infected for 24 hours in the presence of polybrene (2.5 μg/ml). After infection, cells were selected with 0.5 μg/ml puromycin for 10 days. The ORFs of SMAD2 and SMAD3 generated by PCR amplification were cloned into pcDNA 3.1 (Invitrogen; Life Technologies). The 3′UTRs of SMAD2 and SMAD3 were respectively amplified and cloned downstream to the luciferase gene in a modified pGL3 control vector. Antagomir-323-3p was purchased from RIBOBIO Company (Guangzhou, China). The TGF-b luciferase reporter gene plasmid p3TP-lux was a gift from Joan Massague & Jeff Wrana (Addgene plasmid # 11767).

### RNA extraction, reverse transcription, and real-time RT-PCR

Total RNA was extracted from cultured cells using the mirVana miRNA Isolation Kit (Ambion; Life Technologies). cDNA was synthesized from total RNA with the TaqMan MicroRNA Reverse Transcription Kit (Applied Biosystems; Life Technologies). Expression of miRNAs was analyzed by real-time PCR using the TaqMan MicroRNA Assay kit (Applied Biosystems; Life Technologies).

### Western blot analysis

Western blot analysis was performed according to a standard method, as described previously [46], using anti-E-cadherin, anti-a-catenin, anti-vimentin and anti-N-cadherin (Bioworld Technology, Louis Park, MN, USA), anti-SMAD2, anti-p-Smad2; anti-SMAD3 and anti-p-Smad3 (Abcam, Cambridge, MA, USA). To control sample loading, the blotting membranes were stripped and re-probed with an anti–α-tubulin or anti-p84 antibody (1:2000, Sigma, Saint Louis, MO, USA). Nuclear extracts were prepared using the Nuclear Extraction Kit (Active Motif), following the manufacturer's instructions.

### Wound healing assay

Cells were seeded on six-well plates and grown to monolayer confluency. Straight wounds were created in the cell monolayers using a sterile pipette tip. Progression of migration was observed and photographed at 24 hours after wounding.

### Cell invasion assay

Cell invasion assay was analysed using the Transwell chambers (Costar; Corning Inc.) coated Matrigel (BD Biosciences). Cells (1 × 10^4^) were plated on upper chamber and the lower chamber was filled with 500 μl DMEM supplemented with 10% FBS. After 24 hours of incubation, cells invading into the bottom side of the filter were fixed in 1% paraformaldehyde, stained with hematoxylin, and counted (Ten random 200× fields per well). Three independent experiments were performed and the data are presented as mean ± standard deviation (SD).

### Luciferase reporter assay

Cells (2× 10^4^) were seeded in triplicate in 24-well plates and incubated for 24-hours. Luciferase reporter plasmids plus 3 ng pRL-TK Renilla plasmid were transfected into the cells using Lipofectamine 2000 Reagent (Life Technologies, USA). After treatment with human recombinant TGF-b1 (R&D Systems, Minneapolis, MN, USA) at 10 ng/ml for 24hrs, cells were harvested and Dual Luciferase Reporter Assay (Promega, USA) was performed according to the manufacturer's instructions.

### Animal study

BALB/c-nu mice (5–6 weeks of age, 18–20 g) were purchased from the Centre of Experimental Animal of Guangzhou University of Chinese Medicine. All experimental procedures were approved by the Institutional Animal Care and Use Committee of Sun Yat-sen University. Mice were randomly divided into 4 groups (n=6 per group). Two groups were injected with miR-323-3p-transduced PANC-1 cells and the control cells via lateral tail veins. Another two groups were intravenously injected with PANC-1 cells in the mice, followed by a succession of either control antagomir or antagomir-323-3p treatments via the tail vein, twice per week for 3 weeks. Tumor colonization and growth in the lung were detected by an IVIS imagining system (Caliper). Mice were sacrificed on day 40 after injection. Lungs were fixed in formalin and embedded in paraffin using the routine method. H&E staining was performed on sections from paraffin-embedded samples for histological examination. The number of lung metastasis foci in each group was counted under a low power field.

### miRNP immunoprecipitation

Cells were co-transfected with HA-Ago1 together with 100nM miR-323-3p, followed by HA-Ago1 immunoprecipitation using HA antibody. Real-time PCR analysis of the IP products was used to test the association of the mRNA of SMAD2 and SMAD3 with the RISC complex.

### Statistical analysis

All statistical analyses were carried out using SPSS 18.0 statistical software. Survival curves were plotted using the Kaplan–Meier method and compared by log-rank test. Comparisons between 2 groups were performed using the Student's t test. The nonparametric Mann Whitney test was used to compare the expression levels of miR-323 between two disproportionate groups (172 tumor tissues with 4 normal tissues). In all cases, P < 0.05 was considered statistically significant.

## SUPPLEMENTARY FIGURES AND TABLES


