# Supplementary material for: MicroRNA-323-3p inhibits cell invasion and metastasis in pancreatic ductal adenocarcinoma via direct suppression of SMAD2 and SMAD3
Source: Oncotarget. 2016 Feb 18;7(12):14912–24. doi: 10.18632/oncotarget.7482 (PMC4924761; doi:10.18632/oncotarget.7482)
Supplement: Supplementary file 1 [file oncotarget-07-14912-s001.pdf]

SUPPLEMENTARY FIGURES AND TABLES

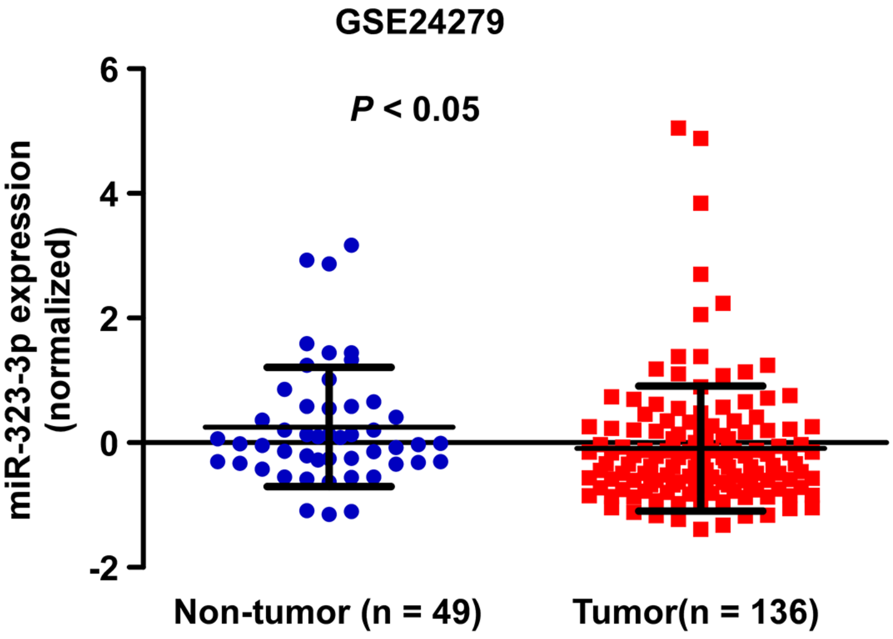

Supplementary Figure S1: Expression profiling of miR-323-3p in PDAC tissues (T; n = 136) and normal pancreatic tissues (N; n = 49) based on microarray datasets from GEO datasets (GSE24279).

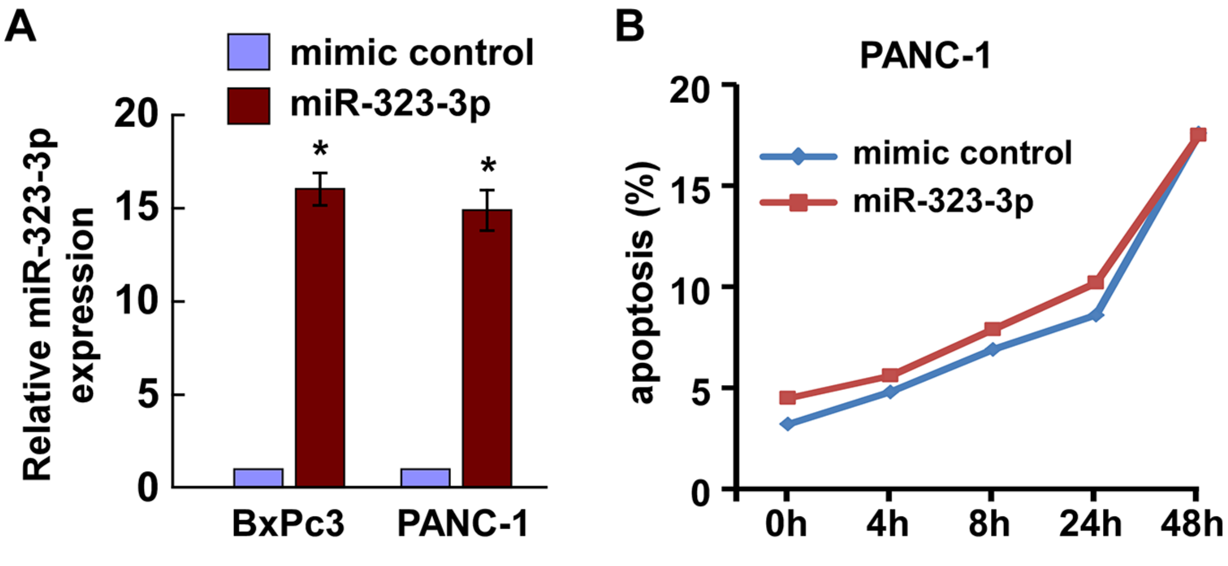

Supplementary Figure S2: A. Relative expressions of miR-323-3p in miR-323-3p-overexpressing pancreatic cancer cells. Each bar represents the mean ± SD of three independentexperiments; *$P < 0.05$. **B.** Cells were transfected with miR-323-3p-overexpressing or vector control construct and plated on poly-HEMA coated plates. Cells were subjected for apoptosis analysis by FACS analysis of Annexin V/PI stained cells. Each bar represents the mean ± SD of three independentexperiments; *$P < 0.05$.

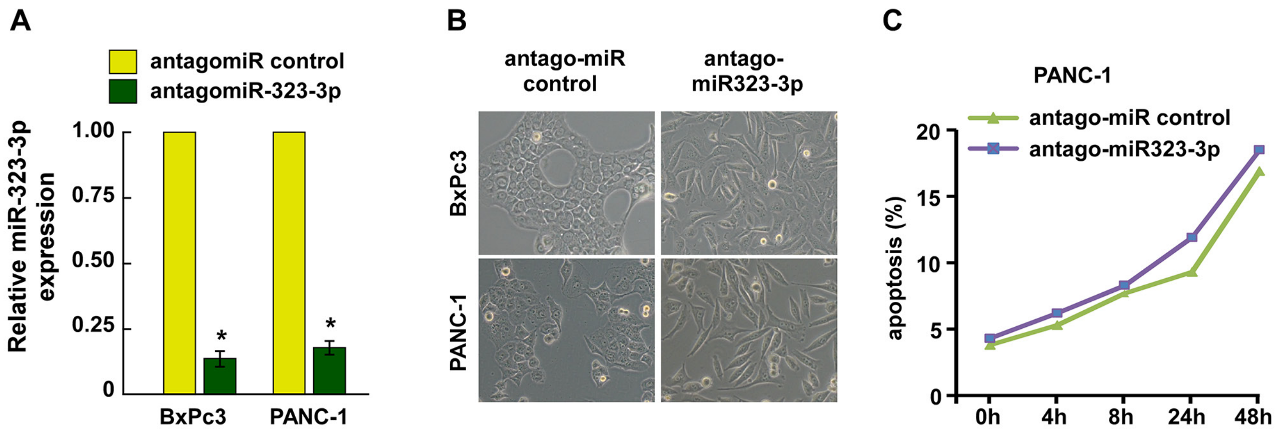

Supplementary Figure S3: A. Relative expressions of miR-323-3p in miR-323-3p-silenced cells or vector control cells examined by Real-Time PCR analysis. **B.** Morphology of PANC-1 and BxPc3 cells expressing either antagomiR-323-3p or vector control are shown by phase contrast. Original magnification, ×400. **C.** Apoptosis analysis by FACS analysis of Annexin V/PI stained cells expressing antago-miR control or antago-miR323-3p. Each bar represents the mean ± SD of three independentexperiments; *$P < 0.05$.

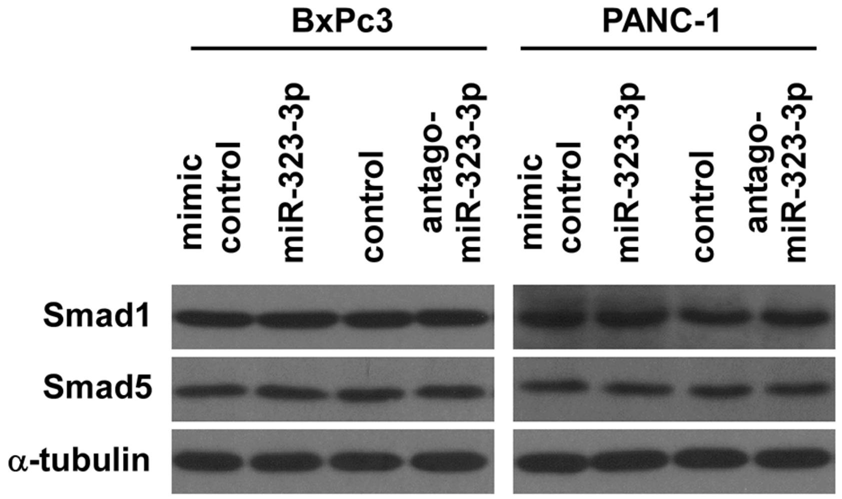

Supplementary Figure S4: Western blotting showing the expression levels of SMAD1 and SMAD5 in the indicated pancreatic cancer cells. α-tubulin was used as a loading control.

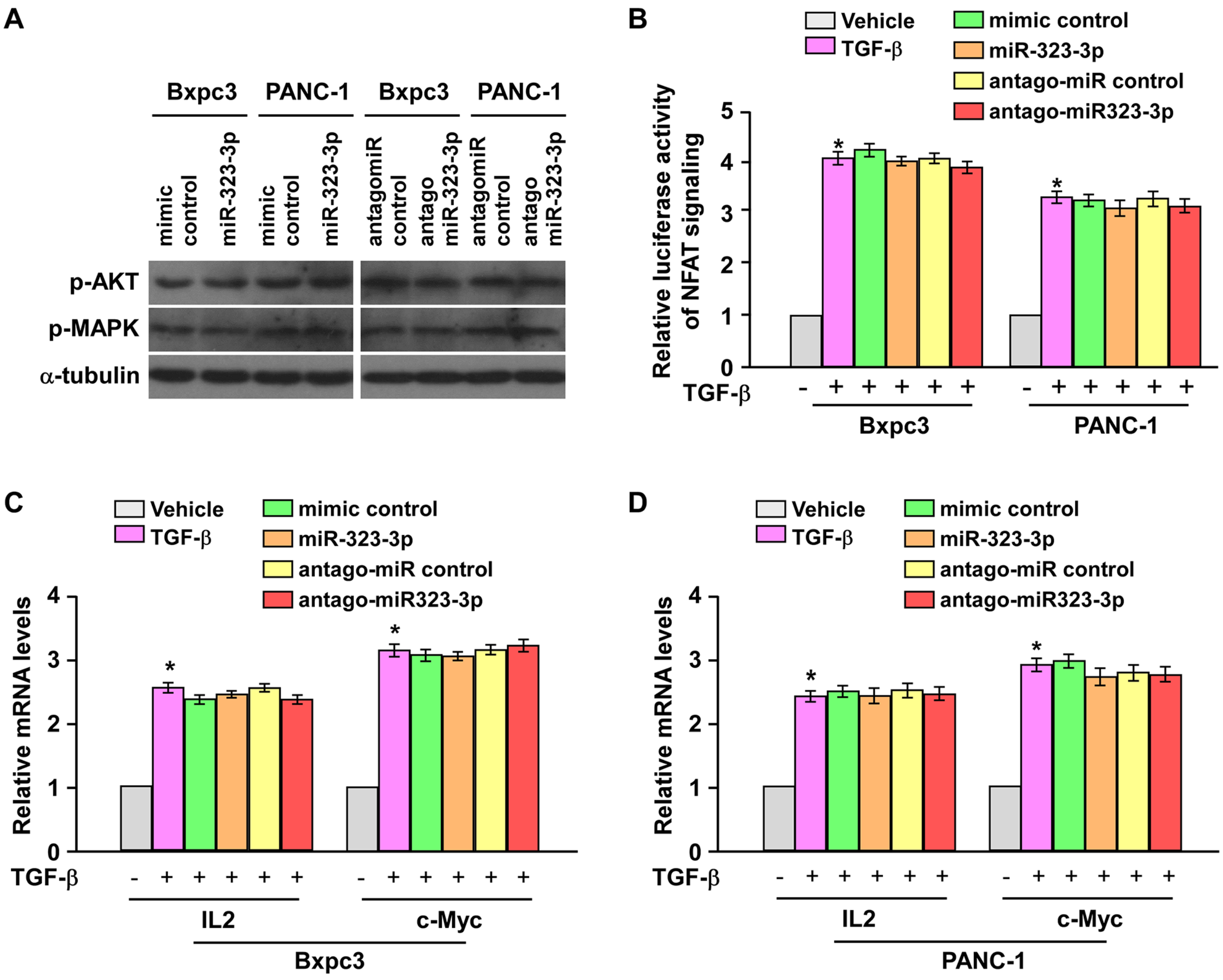

Supplementary Figure S5: A. Western blotting showing the expression levels of phosphorylated AKT (Ser473) and MAPK (Thr180/Tyr182) in the indicated pancreatic cancer cells. α-tubulin was used as a loading control. **B.** The luciferase activities of NFAT signaling in the indicated cells with or without TGF-b treatment. **C.** and **D.** Expression level of IL2 and c-Myc in the BxPc3 cells (C) and PANC-1 cells (D) with the indicated treatments as determined by Real-time PCR analysis. Each bar represents the mean ± SD of three independent experiments; *$P < 0.05$.

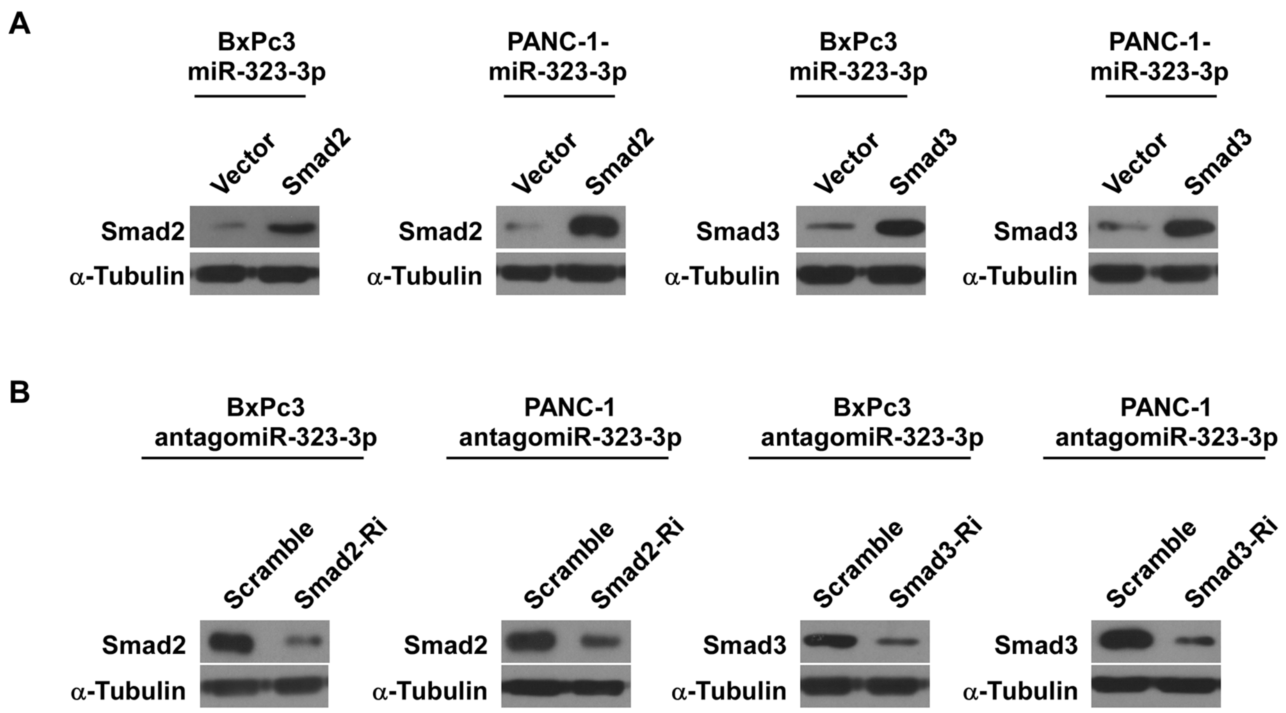

Supplementary Figure S6: Western blotting showing the expression levels of SMAD2 and SMAD3 in the indicated pancreatic cancer cells. α-tubulin was used as a loading control.

Supplementary Table S1: Clinicopathological characteristics and expression of miR-323-3p of 108 pancreatic cancer cases

Characteristics	No.	(%)
Age		
≤mean(65)	57	53
>mean(65)	51	47
Gender		
Male	61	56
Female	47	44
Histologic grade		
G1	14	13
G2	54	50
G3	35	32
G4	5	5
Pathology stage		
I	11	10
II	31	29
III	44	41
IV	22	20
T stage		
1	9	8
2	27	25
3	47	44
4	25	23
N stage		
0	27	25
1	81	75
Distant Metastasis		
No	61	56
Yes	47	44
Vital status		
Alive	74	69
Dead	34	31
Expression of miR-323-3p		
Low (≤median)	54	50
High(>median)	54	50

Supplementary Table S2: Correation between Clinicopathologic Features and miR-323-3p expression in 108 pancreatic cancer tissues

Characteristics	miR-323-3p expression		Pearson Chi-Square	*P* value
	Low	High		
Age			0.037	0.847
≤mean(65)	29	28		
>mean(65)	25	26		
Gender			0.038	0.846
Male	31	30		
Female	23	24		
Histologic grade			1.668	0.644
G1	9	5		
G2	25	29		
G3	18	17		
G4	2	3		
Clinical stage			7.977	0.046
I	3	8		
II	11	20		
III	26	18		
IV	14	8		
T stage			8.501	0.037
1	2	7		
2	9	18		
3	28	19		
4	15	10		
N stage			8.346	0.004
0	7	20		
1	47	34		
Distant Metastasis			10.887	0.001
No	22	39		
Yes	32	15		
Vital status			8.413	0.004
Alive	30	44		
Dead	24	10		
